# Disease of the Lungs

**Published:** 1901-03-30

**Authors:** 


### DISEASE OF THE LUNGS.

**Ætiology.**—Weber[1] reviews two hundred cases of pneumonia. He notes a marked susceptibility of certain families. The left lung is twice as often affected by croupous pneumonia than the right. Influenza was the commonest antecedent in secondary pneumonia. The general mortality, comprising the extremes of life almost entirely, was 9 per cent. He recommends in the first stage, counter-irritants, arterial sedatives, and anodynes: in the infiltration and hepatization stage carbonate and iodide of ammonium and later strychnine.

**Diagnosis.**—Collier[2] gives two cases of diaphragmatic pleurisy simulating perforation of the stomach. After symptoms of dyspepsia, which also might have pointed to gastric ulcer, acute abdominal pain suddenly ensued, with every appearance of perforation. On careful examination, however, they both gave evidence of pleuritic friction, and a subsequent onset of pneumonia.

Abrams[3] describes methods for eliciting pleural friction sound. When râles and rhonchi obscure it, the former may be removed by the inhalation of amyl nitrite. The friction sound may be removed by an assistant fixing the lower part of the chest with his hands. Three other original suggestions he makes with regard to the diagnosis:—

(1) On raising the extended arm up to the head after inspiration, or after full expiration, the parietal pleura is moving in an opposite direction to the visceral, and thus auscultation will reveal any friction.

(2) If the patient lies down for a few minutes on the affected side, and then rises quickly and suspends respiration, the two layers of pleura which are approximated by the action will alter their position.

(3) Pressure with the stethoscope in an intercostal space will approximate the pleuræ.

Squire[4] urges in the diagnosis of cases of pleural exudate with the physical sign of pneumonia the following points:—Diminished resonance (flatness) on percussion, with greatly increased resistance, especially if the upper line of the "flatness" follows the curves of Garland and Ellis. Change in the level of flatness or change of position of patient. Skodaic resonance above the flatness, displacement of organs, restricted movement of the diaphragm on the affected side, diminution or absence of breath sounds, vocal fremitus and resonance, and the aspirating needle.

Dieulafoy[5] divides evil-smelling pleurisies into three classes:—

(1) Fetid pleurisy.
(2) Putrid pleurisy.
(3) Gangrenous pleurisy.

The pleurisy is "fetid" when the pus is not due to a putrefactive or gangrenous process. There is no formation of gas, the inoculation of animals does not produce gaseous phlegmon, and culture tubes show neither fermentation nor gas formation. The bacteria found, in addition to the pneumococcus, streptococcus, and B. coli, are some that produce bad odour, and some may produce colour. These fetid pleurisies have the better prognosis, but should be treated by evacuation as rapidly as possible.

Pleurisy is said to be "putrid" when there is gas in the pleura, or gaseous phlegmon is set up along the track of an exploratory puncture, and animals inoculated die of gaseous phlegmonous œdema. There is rapid prostration with syncope and collapse, and the prognosis is of the gravest. The author states that such conditions occur after abdominal or obstetric operations, even though they themselves seem asceptic and successful; also after appendicitis or otitis. He quotes a case of a successful abdominal hysterectomy, followed some weeks later by a putrid pleurisy. This was traced to a forgotten tampon in the vagina, where before had been a fetid discharge. The same bacteria were found in the vagina, sputum, and pleuritic fluid. Although putrid there were no sloughs cast off into the fluid, and no other evidence of gangrene After complete draining the patient recovered.

Putrid pleurisies may have, therefore, an embolic origin, whether the original focus be vagina, appendix, otitis, osteomyelitis, phlebitis, &c., or due to neighbouring inflammation, lung or mediastinum; abdominal lesions, renal, hepatic or subpleural suppurations are also causes; but some causes are unknown. These bacteria are gas forming, and so may produce pneumothorax without a perforation.

Pleurisy is called "Gangrenous" when sloughs are found in the pus of the empyema. There are the same bacterial agents, aero-anærobic, as produce a simple putrid pleurisy. The line of demarcation is not absolute, putrefaction and mortification can be produced together or successively.

Walsham and Beale,[6] in a paper on the value of skiagraphy in the diagnosis of chest disease, state that the coil must be powerful enough to generate a 12 to 14 inch spark. The patient should lie face downwards on a couch, with the plate under the chest. It is always advisable to examine back as well as front with the screen, and it is useful to examine diagonally as well.

The earliest deposits of tubercle do not yield any appreciable shadow, but cases were demonstrated where shadows revealed tubercle before the appearance of any physical sign. Where one lung was diseased an unsuspected focus can sometimes be demonstrated in the other apex as well The shadows of fibroid changes and adhesions, when sufficiently dense to produce any, differ considerably from those produced by tubercle. Cavities show light areas, and emphysema exceptional translucency. Early dry pleuritis show only a faint shadow. Serous effusion causes faint blurring of the rib shadows, but frequently there is a clear line of demarcation to be seen at the upper border. Purulent effusion causes very dark shadows. Pus in a tube shows a line darker than fluid blood or serum and nearly as dark as bone. Thus abscesses throw dark shadows. Tuberculous nodules could be recognised in the kidney. They also testify to the use of the skiagraphy in detecting aneurisms and morbid growths.

[1] Treatment, Nov., 1900. [2] Birm. Med. Rev., Oct. [3] Dublin Med. Rev. Oct. [4] Treatment, Nov., 1900. [5] La Semaine Méd., Oct. 31st. [6] Lancet, Jan. 19th.

